# Capturing the dynamics of pathogens with many strains

**DOI:** 10.1007/s00285-015-0873-4

**Published:** 2015-03-24

**Authors:** Adam J. Kucharski, Viggo Andreasen, Julia R. Gog

**Affiliations:** 1 Centre for the Mathematical Modelling of Infectious Diseases, London School of Hygiene and Tropical Medicine, London, UK; 2 Department of Mathematics and Physics, Roskilde University, 4000 Roskilde, Denmark; 3 Department of Applied Mathematics and Theoretical Physics, Centre for Mathematical Sciences, University of Cambridge, Cambridge, UK

**Keywords:** Transmission model, Evolution, Cross-immunity, Multi-strain pathogens, Influenza, 37N25 Dynamical systems in biology, 92B Mathematical Biology

## Abstract

Pathogens that consist of multiple antigenic variants are a serious public health concern. These infections, which include dengue virus, influenza and malaria, generate substantial morbidity and mortality. However, there are considerable theoretical challenges involved in modelling such infections. As well as describing the interaction between strains that occurs as a result cross-immunity and evolution, models must balance biological realism with mathematical and computational tractability. Here we review different modelling approaches, and suggest a number of biological problems that are potential candidates for study with these methods. We provide a comprehensive outline of the benefits and disadvantages of available frameworks, and describe what biological information is preserved and lost under different modelling assumptions. We also consider the emergence of new disease strains, and discuss how models of pathogens with multiple strains could be developed further in future. This includes extending the flexibility and biological realism of current approaches, as well as interface with data.

## Introduction

Many human pathogens can be categorized into distinct strains, each defined by its antigenic properties (Balmer and Tanner 2011; Grenfell et al. 2004). These infections, which include influenza (Webster et al. 1992; Wilson and Cox 1990), dengue virus (Rothman 2011) and malaria (McKenzie et al. 2008) are responsible for substantial morbidity and mortality each year. Further, prior infection with one strain of a disease may not always protect against another. For instance, as the influenza virus evolves, antibodies generated against a specific past strain become progressively less effective against the current one (Davenport et al. 1953; Potter 1979). This results in a highly complex system, with pathogens interacting through the partial cross-immunity they generate in the host population. Examining the effect of this interaction on disease outbreaks has therefore posed a major challenge, both theoretically and biologically.

Population dynamic models can generate insights into the mechanisms that drive the transmission of an infection, as well as test new hypotheses about evolution and immunity. In this paper, we review current research into diseases with many strains, providing a detailed comparison of available methods. We also aim to identify key topics for future research that will unify recent developments in the field, providing powerful tools with which to understand the evolutionary, epidemiological and immunological dynamics of these diseases.

## Multiple-strain models

### Influenza

Mathematical models have long been used been used to study disease transmission (Kermack and McKendrick 1927) and control (Ross 1911), but the interaction of disease strains through cross-immunity is a more recent area of research. Early models looked at competition between two strains; infection with one strain conferred immunity to the other for the duration of infection. Such models have been implemented in both discrete (Elveback et al. 1964) and continuous-time (Dietz 1979). Following this work, Castillo-Chavez et al. (1989) introduced a model in which one strain could give imperfect cross-immmunity to another. The work was motivated by the dynamics of influenza, and considered two interacting strains.

Although modelling studies have looked at multi-strain pathogens such as *Plasmodium falciparum* (Gupta and Day 1994; Gupta et al. 1994) or *Neisseria meningitidis* (Gupta et al. 1996; Buckee et al. 2008), influenza remains a central focus for theoretical work. As well as its impact on public health, with seasonal epidemics causing substantial morbidity and mortality, the virus undergoes frequent mutations, resulting in rapid turnover of seasonal strains, as well as between-subtype reassortment, which can lead to the occasional emergence of pandemic variants (Webster et al. 1992; Wilson and Cox 1990). Further, multiple influenza infections are possible during an individual’s lifetime, with a host’s history of infection and immunity determining the result of future exposures (Francis 1960). In turn, this collection of varying individual infection histories shapes the dynamics of the disease at the population level. Capturing the behaviour of diseases such as influenza can therefore require the use of a model that accounts for multiple strains.

One of the most detailed—and computationally intensive—of these frameworks is the individual-based model (Bedford et al. 2012; Ferguson et al. 2003; Tria et al. 2005), which tracks the infection history of every host, updating individuals’ immune status as the disease spreads and evolves during a simulation. Alternatively, population models, in which individuals are grouped into compartments, provide a way of exploring disease dynamics that is analytically tractable as well as easier to implement numerically. One of the earliest of these was the susceptible–infective–recovered (SIR) model (Kermack and McKendrick 1927), which considers the proportion of the population susceptible to, infectious with, and recovered from—and hence assumed immune to—a particular infection. However, the SIR model focuses only the dynamics of a single pathogen: it does not account for the evolving nature of the influenza virus. The susceptible-infective-recovered-susceptible (SIRS) model (Pease 1987; Girvan et al. 2002) can incorporate changes in immunity as a result of disease evolution by assuming individuals who are recovered gradually again become susceptible to the current circulating infection. This can be expanded further by including an additional set of ‘cross-immune’ individuals, resulting in the susceptible–infective–recovered–cross-immune (SIRC) model (Casagrandi et al. 2006). Although they can be explored analytically, the SIRS and SIRC models collect all information about population immunity into one or two variables, which means they do record information about the combination of past infections that generated this immunity.

As we move from an individual-based model, which keeps track of both infection and immune history, to a simpler system, we inevitably sacrifice information for tractability. Population models of multiple strains can therefore be classified by the information that they retain, and the information they do not.

### History-based models

#### Two strain model

**Fig. 1:**
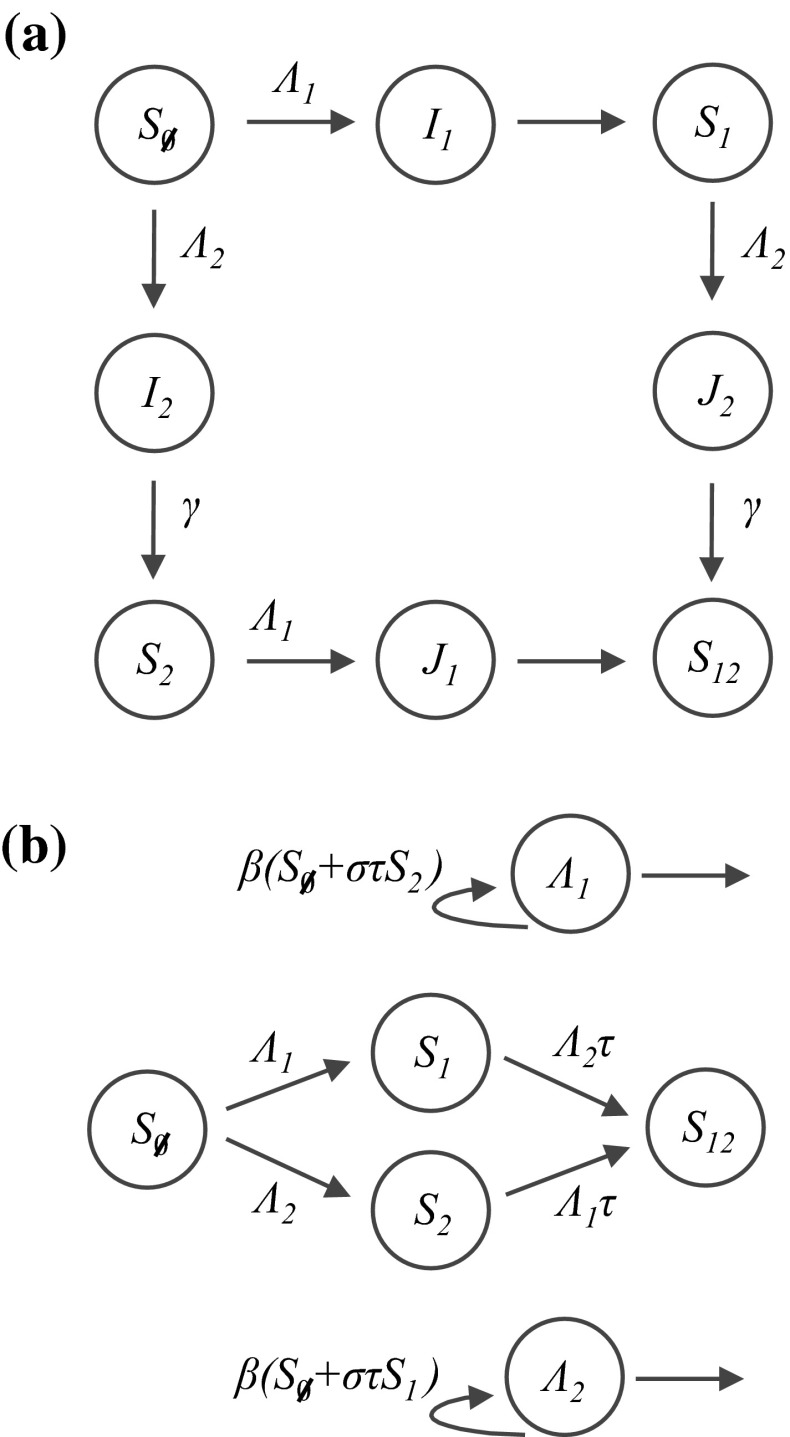
Possible routes of infection in a two strain history-based model. **a** Two strain model in which a host’s new infection history is obtained upon recovery from infection (Castillo-Chavez et al. 1989). $S_i$ denotes the proportion of hosts who have previously been infected—and recovered from—the set of strains $\{i\}$; $I_i$ denotes hosts who are experiencing a primary infection with strain $i$; $J_i$ denotes hosts who are experiencing a secondary infection with strain $i$; $\Lambda _i$ is the force of infection for strain $i$; $\gamma$ is the rate of recovery; and $\tau$ is the relative susceptibility of hosts who have previously been infected with a heterologous strain. Births and deaths are not shown. **b** Two strain model in which infection history obtained immediately upon infection (Andreasen et al. 1997; Gupta et al. 1996). Here $S_i$ denotes hosts who have been infected with the set of strains $\{i\}$, and $\sigma$ denotes the relative infectiousness of hosts who have previously been infected with a heterologous strain

The history-based model is an extension of the SIR model. It has one compartment for each possible combination of prior infection, with cross-immunity dependent on an individual’s infection history. If we assume that immunity is acquired after recovery from infection, we can define a two strain model using eight compartments (Castillo-Chavez et al. 1989): individuals begin in the naive $S_\emptyset$ compartment, which represents the proportion of the population who have never been infected; upon primary infection, they move into $I_i$, where $i \in \{1,2\}$ denotes the infecting strain. Individuals in $I_1$ then recover into the $S_1$ compartment; upon secondary infection with strain $2$, they move into $J_2$; and once recovered from both strains they end up in $S_{12}$. These transitions, and analogous transitions for infection with strain $2$ then strain $1$, are shown in Fig. 1a.

If a host has previously been infected with one strain and is subsequently exposed to another, cross-immunity can be assumed to act in one of—at least—two ways (Andreasen et al. 1997; Gupta et al. 1996): either the host is either less likely to be infected by the second strain (‘reduced susceptibility’), or the host will be less likely to transmit the second strain (‘reduced transmission’). Let $\tau$ denote reduced susceptibility, specifically the relative susceptibility of individuals that have already been infected with one strain. This means the rate at which individuals leave the $S_1$ compartment owing to infection with strain $2$ is $\Lambda _2 \tau S_1$, where $\Lambda _2$ is the force of infection for strain $2$. Next, let $\sigma$ be the relative *infectiousness* of hosts who have previously been infected with the other strain. We define $\beta _i$ to be the rate of transmission for a primary infection with strain $i$. Therefore $\Lambda _2=\beta _2(I_2+ \sigma J_2)$. Finally, we assume that each strain confers total immunity to itself and the population birth/death rate is $\mu$. With these assumptions, the model is as follows,

1 $$\begin{aligned} \frac{dS_\emptyset }{dt} = {}&\mu -[\beta _1 (I_1 + \sigma J_1) + \beta _2 (I_2+ \sigma J_2)] S_\emptyset - \mu S_\emptyset \end{aligned}$$

2 $$\begin{aligned} \frac{dI_1}{dt} = {}&\beta _1 (I_1 + \sigma J_1) S_\emptyset - (\gamma +\mu ) I_1 \end{aligned}$$

3 $$\begin{aligned} \frac{dI_2}{dt} = {}&\beta _2 (I_2 + \sigma J_2) S_\emptyset - (\gamma +\mu ) I_2 \end{aligned}$$

4 $$\begin{aligned} \frac{dS_1}{dt} = {}&\gamma I_1- \beta _1 (I_2 + \sigma J_2) \tau S_1 - \mu S_1 \end{aligned}$$

5 $$\begin{aligned} \frac{dS_2}{dt} = {}&\gamma I_2- \beta _2 (I_1 + \sigma J_1) \tau S_2 - \mu S_2 \end{aligned}$$

6 $$\begin{aligned} \frac{dJ_1}{dt} = {}&\beta _1 (I_1 + \sigma J_1) \tau S_2 - (\gamma +\mu ) J_1 \end{aligned}$$

7 $$\begin{aligned} \frac{dJ_2}{dt} = {}&\beta _2 (I_2 + \sigma J_2) \tau S_1 - (\gamma +\mu ) J_2 \end{aligned}$$

8 $$\begin{aligned} \frac{dS_{12}}{dt} = {}&\gamma (J_1+J_2) - \mu S_{12} \end{aligned}$$

where $\gamma$ is the rate of recovery.

As additional strains are added, the complexity of this model increases substantially. For $n$ strains, the model has $(n+2) 2^{n-1}$ variables (Andreasen et al. 1997). To simplify the model, we can assume that individuals obtain an updated infection history immediately upon infection (Gupta et al. 1996; Ferguson and Andreasen 2002). This is equivalent either to assuming that hosts are immediately available for further infection—and hence superinfection is implicitly allowed—or having a mathematical approximation in which hosts spend a negligibly small part of their lives infected (i.e. $\mu /\gamma$ is small).

We define $\hat{I}_i=I_i+\sigma J_i$ to be the weighted proportion of the population who contribute to the force of infection for strain $i$. Because infection history is updated immediately, individuals who are in the $\hat{I}_i$ compartment are always in one of the $S$ compartments too. Hence $S_\emptyset +S_1+S_2+S_{12}=1$.

Let $\Lambda _i=\beta \hat{I}_i$ denote the force of infection for strain $i$. We can specify ${d \Lambda _{i}}/{dt}$ using the definition of $\Lambda _i$ and Eqs. 3–4 and 6–7. Hence our system no longer depends explicitly on $I_i$ and $J_i$, and we can rewrite the model using $2^{n}+n$ variables. Note that we do not need to keep track of individuals who have left $\hat{I}_i$, as they are already included in one of the $S_i$ compartments. As before, $\tau$ denotes the relative susceptibility of hosts previously infected with the other strain and $\sigma$ denotes relative infectiousness. Hence $\tau$ influences the transitions between the $S$ compartments (Fig. 1b), while both $\tau$ and $\sigma$ influence the rate at which the force of infection changes. The updated system is as follows:

9 $$\begin{aligned} \frac{dS_{\emptyset }}{dt} = {}&\mu - ( \Lambda _1+ \Lambda _2) S_\emptyset -\mu S_\emptyset \end{aligned}$$

10 $$\begin{aligned} \frac{dS_{1}}{dt} = {}&\Lambda _1 S_{\emptyset } - \tau \Lambda _2 S_{1} -\mu S_1 \end{aligned}$$

11 $$\begin{aligned} \frac{dS_{2}}{dt} = {}&\Lambda _2 S_{\emptyset } - \tau \Lambda _1 S_{2} -\mu S_2 \end{aligned}$$

12 $$\begin{aligned} \frac{dS_{12}}{dt} = {}&\tau \Lambda _1 S_2 + \tau \Lambda _2 S_1 -\mu S_{12} \end{aligned}$$

13 $$\begin{aligned} \frac{d \Lambda _{1}}{dt} = {}&\beta (S_\emptyset + \sigma \tau S_2) \Lambda _1 -(\gamma + \mu ) \Lambda _1 \end{aligned}$$

14 $$\begin{aligned} \frac{d \Lambda _{2}}{dt} = {}&\beta (S_\emptyset + \sigma \tau S_1) \Lambda _2 -(\gamma + \mu ) \Lambda _2 . \end{aligned}$$

#### Extension to multiple strains

**Fig. 2:**
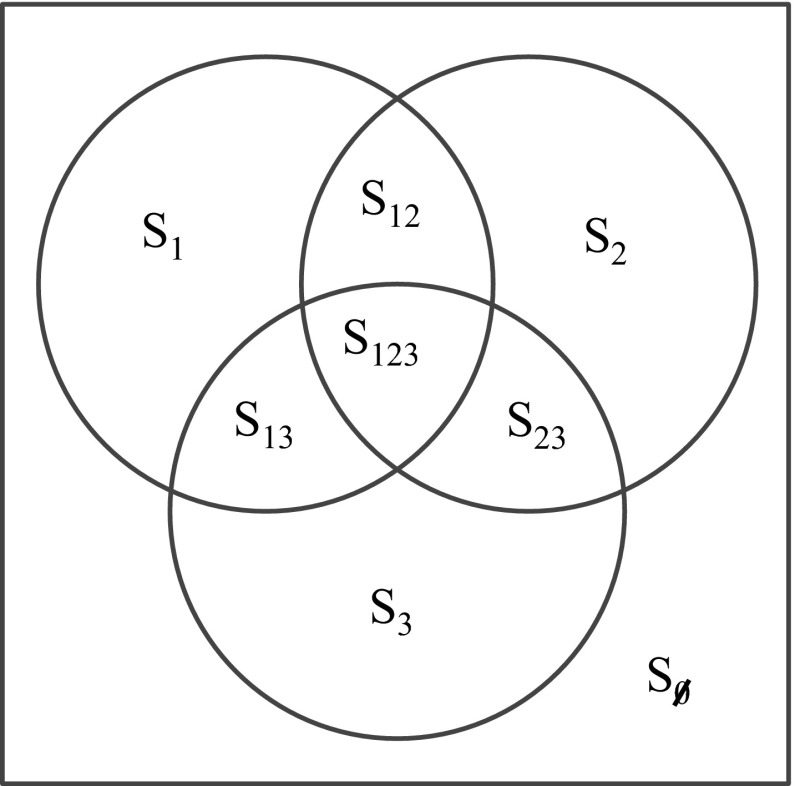
Possible infection histories in a three strain model. Sets are disjoint, with subscripts indicating which collection of strains have previously been seen

When multiple strains are included in a model, set notation can be used to distinguish between infection histories (Andreasen et al. 1997). For $n$ disease strains, we define $\mathcal {N}=\{1,\ldots ,n\}$ to be the set of all strains, and let $X$ be some subset of $\mathcal {N}$. There are $2^n$ subsets of $\mathcal {N}$, each representing a different infection history (including the empty set $\emptyset$ for totally naive individuals). The possible subsets for a three strain model are shown in Fig. 2. $S_X$ denotes the proportion of the population that have been infected by all strains in set $X \subseteq \mathcal {N}$, but not by any strain in $\mathcal {N} {\setminus } X$ (i.e. strains not in their infection history). These sets are disjoint and $\sum _{X \subseteq \mathcal {N}} S_X=1$.

To include the reduced transmission assumption, we define $\sigma (Y,i)$ to be the relative contribution to the force of infection for strain $i$ from individuals who have infection history $Y$. We require $\sigma (\emptyset ,i)=1$ for any $i$, so total lack of acquired immunity results in transmission at rate $\beta$, and $\sigma (Y,i)=0$ if $i \in Y$, to prevent transmission of an already seen strain.

As before, reduction in susceptibility acts in two ways. First, individuals with infection history $Y$ have the force of infection of strain $i$ reduced by a factor $\tau (Y,i)$. Second, the rate at which $\Lambda _i$ increases is also reduced by $\tau (Y,i)$, due to reduced disease prevalence among hosts in $S_Y$. If we assume that infecting strains immediately become part of an individual’s infection history (Anderson and May 1991), we obtain the following set of equations (Ferguson and Andreasen 2002),

15 $$\begin{aligned} \frac{dS_{X}}{dt} = {}&\mu \delta _{X,\emptyset } + \sum _{j \in X} \tau (X {\setminus } j,j) \Lambda _j S_{X {\setminus } j} - \sum _{j \notin X}\tau (X,j) \Lambda _j S_{X} -\mu S_X \end{aligned}$$

16 $$\begin{aligned} \frac{d \Lambda _{i}}{dt} = {}&\beta \Lambda _i \sum _{Y \subset \mathcal {N}} \tau (Y,i) \sigma (Y,i) S_Y -(\gamma + \mu ) \Lambda _{i} \end{aligned}$$

where $\mu$ represents population birth and death rate (assuming constant population size), $\delta _{X,\emptyset }$ is the Kronecker delta function (hence individuals are born only into the $S_\emptyset$ compartment), and $\gamma$ denotes recovery rate.

### Model dimension

The drawback with history-based models is the sheer number of possible variables that the system generates: given $n$ strains, there are $2^n$ combinations of infection an individual could have seen (Andreasen et al. 1997). Although the dynamics of up to ten strains have been examined using a full history-based framework (Gomes et al. 2002), it has been technically challenging to explore more strains than this.

One alternative is to focus on the equilibrium dynamics of a completely symmetric system, where all strains have the same epidemiological properties (Abu-Raddad and Ferguson 2004). Models with this very specific structure are not intended as a system to be fitted to disease data, but rather make it possible to make some analytic progress on the problem of strain complexity by exploring symmetric extreme cases. Alternatively, reduced versions of the history-based model, described in the following sections, can be used to explore certain aspects of the system in a tractable way.

### Model reduction via symmetry

It is possible to use the symmetry of the strain space to reduce the number of variables in Eqs. 15–16. For instance, suppose there are three strains, with strains 1 and 3 giving the same degree of cross-immunity to strain 2, but no cross-immunity to each other. If strains 1 and 3 have the same epidemiological properties, they can therefore be recorded as one variable in the system (Lin et al. 1999).

Next, suppose each strain is defined by two loci and two alleles, which is analogous in terms of symmetry to a circle of four strains (Gog and Swinton 2002). If cross-immunity only acts to reduce transmission (i.e. $\tau \equiv 1$), then Gupta et al. (1998) showed that symmetry of this strain space can be exploited to define the model using only 8 immunity variables, rather than $2^4=16$. This approach was an extension of an earlier, approximate method, which used four immunity variables (Gupta et al. 1996). The approach can also be extended for strains with multiple alleles (Gupta et al. 1998).

The same type of reduction may be exploited more generally if we assume cross-immunity between strains—as measured by antigenic similarity—is consistent with a space in which a given set of strains can be organized into antigenic ‘neighbourhoods’ (Ferguson and Andreasen 2002). For each strain $i$, define $\{i\} = N_0(i) \subseteq N_1(i) \subseteq \dots \subseteq N_m(i)=\mathcal {N}$ to be a nested sequence denoting collections of strains that are within a particular antigenic distance of strain $i$. At one end, we have the strain $i$ itself, $\{i\}$; at the other, the complete set of all strains $\mathcal {N}$.

**Fig. 3:**
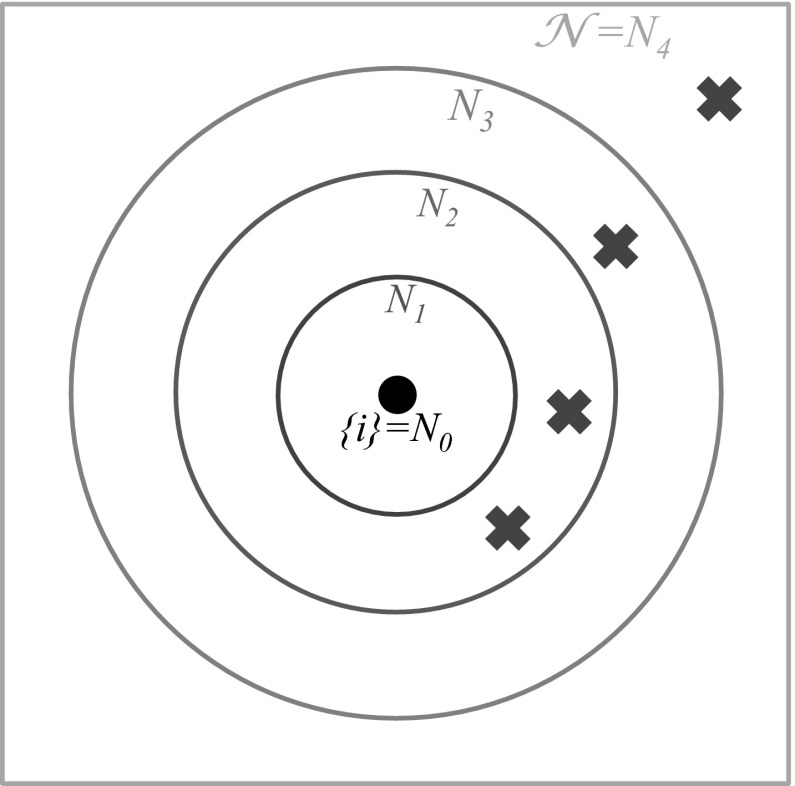
Example of cross-immunity in nested model. *Circles* show antigenic neighbourhoods for strain $i$, indicated by *black dot*. *Blue crosses* show strains in infection history. As the nearest strains are in the set $N_2$ but not in $N_1$, cross-immunity would be equal to $\sigma _2$ (colour figure online)

Given a particular infection history, we require cross-immunity against strain $i$ to depend on the distance between past infections and strain $i$. If a host’s most related previous infection is in the set $N_k(i){\setminus } N_{k-1}(i)$, then we assume transmission is reduced by a factor $\sigma _k$ (Fig. 3). Previously seen strains that are less related, and hence in larger neighbourhoods, do not influence cross-immunity. Hence this formulation is equivalent to cross-immunity taking the form of a minimum function, with $\sigma (Y,i)= \sigma _k$, where

17 $$\begin{aligned} k={}&\min \{j ~|~ Y \cap \left( N_j(i){\setminus } N_{j-1}(i) \right) \ne \emptyset \}. \end{aligned}$$

If this form holds, population immunity to strain $i$ can be expressed in terms of the number of hosts with immunity to one or more strains in each of the $k=0,\dots ,m$ neighbourhoods,

18 $$\begin{aligned} \hat{S}_i^k=\sum _{N_k(i) \cap X \ne \emptyset } S_X \end{aligned}$$

where $\hat{S}_i^m=1$ by definition. If we assume reduced transmission (i.e. $\tau \equiv 1$), Eqs. 15–16 can be written as follows (Ferguson and Andreasen 2002)

19 $$\begin{aligned} \frac{d\hat{S}_i^k}{dt} = {}&\left( \sum _{j \in N_k(i)} \Lambda _j \right) (1-\hat{S}_i^k)- \mu \hat{S}_i^k \end{aligned}$$

20 $$\begin{aligned} \frac{d \Lambda _{i}}{dt} = {}&\beta _i \Lambda \sum _{k=1}^m \sigma _k (\hat{S}_i^k-\hat{S}_i^{k-1}) - (\gamma + \mu ) \Lambda _{i} . \end{aligned}$$

(Note that Ferguson and Andreasen (2002) used $T_i^k$ rather than $\hat{S}_i^k$.) For $n$ strains with $m$ immunity neighbourhoods, this method reduces the $\mathcal {O}(2^n)$ system to $n \times (m+1)$ dynamic equations. For strains defined by $E$ epitopes, each taking one of $A$ alleles, and immunity depending on the number of epitopes previously seen, there are therefore $A^E(E+1)$ equations (Minayev and Ferguson 2008).

Reduction in model dimension via symmetry requires three main sacrifices to be made. First, cross-immunity must take the form of reduced transmission; a similar general reduction is yet to be achieved for a model with reduced susceptibility. The cross-immunity function is also constrained by the set of antigenic neighbourhoods, with the reduction dependent on the use of a minimum function, rather than a generic $\sigma (Y,i)$ cross-immunity term. Finally, without information on individuals’ full infection histories, it is not possible to ascertain the level of immunity against new strains. For instance, suppose a novel strain $z$—not previously included in Eqs. 19–20—were introduced to the population. Using the information stored in the $2^n$ variables, it would be possible, via the $\sigma (Y,z)$ function, to calculate susceptibility to the new strain in the full history based model. However, in this reduced framework there is no way of constructing the new $\hat{S}_z^k$ compartments from the existing set of variables, $\{ \hat{S}_i^k ~|~ i \in \mathcal {N}, k=0,\dots ,m\}$.

### Model reduction via age structure

Another way to group strains into overlapping sets, as in Eq. 18, is to define $\hat{S}_i$ to be the proportion of susceptibles who have seen *at least* strain $i$. Formally,

21 $$\begin{aligned} \hat{S}_{i} = \sum _{X | i\in X} S_X. \end{aligned}$$

If we assume reduced transmission again, Eqs. 15–16 can therefore be expressed as (Kucharski and Gog 2012a),

22 $$\begin{aligned} \frac{d S_\emptyset }{d t}= {}&\mu -S_\emptyset \sum _i \Lambda _i -\mu S_\emptyset \end{aligned}$$

23 $$\begin{aligned} \frac{d \hat{S}_i}{d t}= {}&(1-\hat{S}_i) \Lambda _i -\mu \hat{S}_i \end{aligned}$$

24 $$\begin{aligned} \frac{d \Lambda _{i}}{d t} = {}&\beta Q_i \Lambda _i -(\gamma + \mu ) \Lambda _{i} \end{aligned}$$

where $Q_i$ is the ‘potential infectivity’ of strain $i$ (Andreasen and Sasaki 2006),

25 $$\begin{aligned} Q_i=\sum _{Y \subset \mathcal {N}} \sigma (Y,i) S_Y . \end{aligned}$$

This measures the contribution to the force of infection if all hosts were to become infected.

Although Eqs. 23–24 only depend on $i$ explicitly, $Q_i$ is a function of all $2^n$ sets, and unless each $S_Y$ can be expressed in terms of $\hat{S}_i$ variables, the system is intractable. One solution is to introduce age structure (Kucharski and Gog 2012a).

If immunity acts only to reduce transmission, one might naively expect the probability of having been infected with any two particular strains to be independent: infection with the first strain will not change the rate at which hosts become infected with the second, just the rate at which they transmit. Hence in a two-strain model, might expect $S_{12}=S_1 S_2$. However, if we know a randomly chosen host has previously been infected with the first strain some point in their life, it means they are more likely to be old than young. Hence they are more likely to have also experienced another specific event in the past, such as infection with the second strain. This means $S_{12} \ge S_1 S_2$.

The problem can be resolved using the same age-structured logic; if we focus on a specific age group, independence is maintained. In the full model, we can therefore express the proportion of the population with a particular infection history in terms of the component parts of that infection history:

26 $$\begin{aligned} \frac{S_Y(a,t)}{P_a}= \prod _{j \in Y} \frac{\hat{S}_j(a,t)}{P_a} \prod _{k \notin Y} \left( 1- \frac{\hat{S}_k(a,t)}{P_a} \right) \end{aligned}$$

where $P_a$ as the equilibrium age distribution of the population. This makes it possible to express $Q_i(a)$ as a function of the variables $\{ \hat{S}_j(a) ~|~ j \in \mathcal {N} \}$. Using this result, we obtain an $\mathcal {O}(n)$ model:

27 $$\begin{aligned} \frac{\partial S_\emptyset }{\partial t}= {}&\mu \delta (a)-S_\emptyset \sum _i \Lambda _i -\mu S_\emptyset -\frac{\partial S_\emptyset }{\partial a} \end{aligned}$$

28 $$\begin{aligned} \frac{\partial \hat{S}_i}{\partial t}= {}&(P_a-\hat{S}_i) \Lambda _i -\mu \hat{S}_i -\frac{\partial \hat{S}_i}{\partial a} \end{aligned}$$

29 $$\begin{aligned} \frac{d \Lambda _{i}}{d t} = {}&\left( \int _0^\infty \beta Q_i(a) ~da \right) \Lambda _i -(\gamma + \mu ) \Lambda _{i}. \end{aligned}$$

where $\delta (a)$ is the Dirac delta function. This method of reduction, using independence to reconstruct the $S_Y$ sets, avoids two of the drawbacks of the symmetry approach discussed in the previous section. First, $\sigma (Y,i)$ is no longer restricted to a minimum function, and can now take a variety of forms. Second, because we can reproduce any combination of past infections, it possible to calculate $Q_i$—and hence the level of population immunity—for any strain, even if it was not present in the system initially. The model can therefore incorporate a much larger number of potential strains, and can be used to assess the amount of selection on specific strains of an evolving pathogen.

As well as the necessary reduced transmission assumption, which was also required in the previous section, there are two additional drawbacks to the age-structured approach. The introduction of age dependency increases model complexity, requiring a system of PDEs rather than ODEs, making it challenging to obtain analytic results, and the requirement that infection with each strain is independent for a specific age group also limits the type of population structure that can be imposed. Although Eqs. 28–29 are still valid if more realistic transmission between age groups is introduced (Kucharski and Gog 2012c), a metapopulation framework, for example, with commuting between patches would not be possible because individuals arriving from different subpopulations may have previously been exposed to different strains (Wikramaratna et al. 2014).

### Status-based models

The full individual-based model records both the infection history and current immune status of each host. However, there may not be a straightforward relationship between the two: for influenza, infections may not always produce an immune response, and immunity to a certain strain could potentially be generated by one of several past infections (Potter 1979). In principle, it should be possible to develop a compartmental model that accounted for both infection history and immune status. However, in practice the number of possible combinations of infection history and immune status—and hence compartments required—would likely result a model more complex than even a full individual-based framework. To ensure analytical and computational tractability, history-based models therefore capture the individual infection histories in a population, but not the immune statuses; status-based models (Gog and Swinton 2002) do the opposite, recording the current immune status of individuals in the population, but not the combination of past infections that generated that immunity.

In a history-based framework, partial cross-immunity must take form of every individual being equally partially immune to a particular strain. In other words, it is assumed that individuals with the same infection history will respond to subsequent infection in an identical way: if the set of strains $Y$ have been previously seen, they will transmit strain $i$ with probability $\sigma (Y,i)$.

An alternative assumption is that upon infection some individuals become completely immune, while the rest remain susceptible. This is known as ‘polarized immunity’ (Gog and Swinton 2002). It is possible to include this assumption if the model is ‘status-based’, with compartments that represent which strains an individuals is totally immune to. The assumption of polarized immunity is not essential in a status-based model (see below), although it does serve to give perhaps the simplest verbal interpretation of the system and also illustrates a type of model that cannot be captured in a history-based framework.

First, we describe the full status-based model. Suppose $S_X$ now represents the proportion of individuals who are immune to strains in set $X$. Define $C(Y,X,j)$ to be the probability an individual who previously had immunity to a set of strains $Y$ gains immunity against the set of strains $X$ upon infection with strain $j$. Assuming that cross-immunity acts to reduce susceptibility, the full status-based model can be expressed as follows (Gog and Swinton 2002),

30 $$\begin{aligned} \frac{dS_{X}}{dt} = {}&\mu \delta _{X,\emptyset } + \sum _{j \in \mathcal {N}} \sum _{Y \subset \mathcal {N}} C(Y,X,j) \Lambda _j S_{Y}- \sum _{j \notin X} \Lambda _j S_{X} -\mu S_X \end{aligned}$$

31 $$\begin{aligned} \frac{d \Lambda _{i}}{dt} = {}&\beta _i \Lambda _i \sum _{Y | j \notin Y} S_Y -(\gamma + \mu ) \Lambda _{i} \end{aligned}$$

It is assumed that hosts are susceptible to the infecting strain, that each strain gives immunity to itself, and immunity is only gained. In Eqs. 30–31, this means that $C(Y,X,j)$ takes non-zero values only if $j \notin Y$, $j \in X$ and $Y \subset X$ (Gog and Swinton 2002).

Once again, if cross-immunity is assumed to reduced transmission rather than susceptibility, a reduced version of the status-based model can be expressed in terms of the proportion of the population susceptible to each particular strain (Gog and Grenfell 2002),

32 $$\begin{aligned} \theta _i=1-\sum _{i \in X} S_X. \end{aligned}$$

The full system is as follows (Gog and Grenfell 2002),

33 $$\begin{aligned} \frac{d \theta _{i} }{dt} = {}&\mu -\theta _i \sum _j \beta \sigma _{ij} I_j -\mu \theta _{i} \end{aligned}$$

34 $$\begin{aligned} \frac{dI_{i}}{dt} = {}&\beta \theta _i I_i - \gamma I_i -\mu I_i. \end{aligned}$$

where $\sigma _{ij}$ is the probability an individual will leave compartment $\theta _i$ upon infection with strain $j$. Although the assumption of reduced transmission is important, it is possible to collapse the system to $\mathcal {O}(n)$ because the equations track the immune status of the population in a minimal way. Only the information essential for knowing how strain prevalences change in future is retained.

In an equivalent interpretation of Eqs. 33–34, the variable $\theta _i$ can be thought of as tracking the ‘effective susceptibility’ of the population to strain $i$. Under the reduced transmission assumption, this means the contribution to force of infection if the full population were infected.

Such an interpretation includes the possibility that individuals are partially immune, and hence upon infection would transmit at a lower rate. These individuals are represented in the model by being partially in the $\theta _i$ compartment (Gog 2008). In the reduced transmission status-based model, $\theta _i$ can therefore be thought of as a sum of the population proportions weighted by their relative transmission potential for strain $i$.

As well as deriving an $\mathcal {O}(n)$ model using the reduced transmission assumption, it is possible to simplify the model in Eqs. 30–31 to an $\mathcal {O}(n^2)$ framework through a moment-closure approximation (Kryazhimskiy et al. 2007). This is implemented by expressing higher order terms as a combination of lower order ones. For example, an order-1 approximation reduces the model so that the dynamics are expressed in terms of $\hat{S}_{i}$, the proportion of the population that have immunity against strain $i$, where

35 $$\begin{aligned} \hat{S}_{i} = \sum _{X | i\in X} S_X. \end{aligned}$$

If we assume that individuals have probability $\sigma _{ij}$ of gaining immunity to strain $i$ upon infection with strain $j$, we can set $C(Y,X,j)=\sigma _{ij}$ if $i,j \notin Y$, and zero otherwise. Rewriting Eqs. 30–31 using Eq. 35, we obtain (Kryazhimskiy et al. 2007) :

36 $$\begin{aligned} \frac{d\hat{S}_{i}}{dt} = {}&\sum _{j \in \mathcal {N}} \Lambda _j \sigma _{ij} (1-\hat{S}_{i}-\hat{S}_{j}+\hat{S}_{ij})-\mu \hat{S}_{i} \end{aligned}$$

37 $$\begin{aligned} \frac{d \Lambda _{i}}{dt} = {}&\beta _i \Lambda _i (1-\hat{S}_{i}) -(\gamma + \mu ) \Lambda _{i}. \end{aligned}$$

Note that $(1-\hat{S}_{i}-\hat{S}_{j}+\hat{S}_{ij})$ is the proportion of the population that do not have immunity against strain $i$ or $j$. For the model to be order-1, we need to express $\hat{S}_{ij}$, the proportion of the population that have immunity against strains $i$ and $j$, in terms of $\hat{S}_{i}$ and $\hat{S}_{j}$. One option is to use independence closure, with higher order terms approximated as follows,

38 $$\begin{aligned} \hat{S}_{ij} = \left\{ \!\! \begin{array}{l@{\quad }l} \hat{S}_{i}\hat{S}_{j} &{} \text {if } i \ne j \\ \hat{S}_{i} &{} \text {if }i=j \end{array} \right. . \end{aligned}$$

### Comparison of models

#### Model structure

In mathematical modelling of biological systems, there is often a need to balance complexity, particularly the number of variables in a model, with the ability to include biologically realistic assumptions. When evaluating strain models here, we also consider whether a model can incorporate the addition of new strains mid-way through a simulation without recording additional variables in advance.

The main compartmental models currently available for exploring multiple strain dynamics are summarised in Table 1. Many of the models with few variables require that cross-immunity between strains acts to reduce transmission. The assumption of reduced transmission is mathematically convenient because it means immunity to one strain does not influence susceptibility to another. Hence immunity will only change the rate at which individual passes the infection on, and not their probability of being infected. If cross-immunity leads to a reduction in susceptibility, the crucial simplifications in Eqs. 19, 23 and 33 are no longer possible.

**Table 1:** Comparison of different models for $n$ strains

Model	Type	Variables	New strains	Immunity reduces
Individual-based model	–	Many	Yes	Susceptibility/transmission
Andreasen et al. (1997)	HB	$\mathcal {O}(2^n)$	Yes	Susceptibility/transmission
Gupta et al. (1998)$^*$	HB	$\mathcal {O}(n)$	No	Transmission
Kucharski and Gog (2012a)	HB	$\mathcal {O}(n)$	Yes	Transmission
Gog and Swinton (2002)	SB	$\mathcal {O}(2^n)$	No	Susceptibility
Gog and Grenfell (2002)	SB	$\mathcal {O}(n)$	No	Transmission
Kryazhimskiy et al. (2007)	SB	$\mathcal {O}(n^2)$	No	Susceptibility/transmission

Only two models store enough information to permit the introduction of new strains

*HB* history-based, *SB* status-based

$^*$ Generalised by Ferguson and Andreasen (2002)

From a biological point of view the assumption of reduced transmission can be awkward (Ballesteros et al. 2009; Kryazhimskiy et al. 2007). This is because upon infection we expect two events: the host becoming ill and transmitting the disease, and the production of antibodies by host’s immune system. If the host already has immunity to that strain, their current antibodies might block infection without transmission or production of new antibodies occurring. Under the reduced infectivity assumption, immunity prevents an infected host from transmitting the virus, but does not prevent additional gain of immunity. This could lead to an overestimate of population immunity (Ballesteros et al. 2009). Despite this potential caveat, however, the dynamics of the history-based model appear to relatively insensitive to whether immunity is assumed to reduce transmission or susceptibility (Ferguson and Andreasen 2002).

There is also the issue of whether cross-immunity is more plausible as it appears in a history-based model, or a status-based model with polarised immunity. Comparing to model output with the observed evolutionary dynamics of influenza can provide some insights (Ballesteros et al. 2009). Although it has been suggested that antigenic cluster replacement cannot occur in a simple SIR model (Gökaydin et al. 2007), the results of Ballesteros et al. (2009) imply that it is possible in a status-based model with reduced transmission, but not in reduced susceptibility models, or a reduced transmission history-based model: in these, punctuated antigenic evolution results in too high a depletion of susceptibles. In addition, there appears to be a fundamental difference in the dynamics of the status-based and history based-models, with oscillations absent in the status-based framework (Dawes and Gog 2002). The precise assumptions that lead to oscillations in different strain models are yet to be established, however, and the determining factors in a mathematical framework may not have comprehensible analogues in our interpretations of the ‘biology’ of the model (Dawes and Gog 2002).

These discrepancies illustrate the importance of understanding how different assumptions about cross-immunity affect model outputs. Biologically plausible assumptions do not necessarily generate biologically plausible dynamics, and vice versa. Moreover, choosing between two biologically distinct assumptions—such as reduced susceptibility and transmission—can sometimes have a negligible effect on model dynamics. Strain models inevitably have to balance realism with tractability; it is therefore important to know how different simplifications and assumptions influence model predictions.

In some cases, it is possible to incorporate additional realism without substantial additional complexity. History-based models require that all individuals with a particular infection history respond to a new strain in the same way. In contrast, status-based models using polarised immunity can include heterogeneity in immune response between individuals, as can individual-based models. For history-based models, one way to incorporate heterogeneity is to explicitly group hosts by a characteristic such as genotype (Gupta and Galvani 1999). Alternatively, if the characteristic of interest is age, differences could be explored in an age-structured reduced model: individuals are already grouped by age, and cross-immunity could therefore be defined as a function of age as well as infection history. Such a framework could be used to explore the phenomenon of ‘immunosenescence’, whereby the elderly exhibit a weaker immune response than younger groups (Caruso et al. 2009).

Further, in status-based models probability distributions have been used to vary the transition from one immune status to another (Cobey and Pascual 2011). This idea could also be used in history-based models, by treating the cross-immunity parameter as a random variable. There is also potential for this approach to be combined with within-host approximations (Pepin et al. 2010; Volkov et al. 2010). This would allow for more detailed exploration of how assumptions about the immune system affect population level dynamics.

#### Biological applications

As well as depending on the assumptions that can be included, choice of modelling formulation will be influenced by the biological question being addressed. If the aim is to understand how cross-immunity affects the dynamics of the infection, models need to be sufficiently simple to simulate the number of hosts infectious with each strain at each point in time. Knowledge of the precise population history of infection and immunity is not required, as long as disease incidence is recorded. Some studies have used such models to examine the extent of antigenic variation over time, and the antigenic relationship (‘strain structure’) between co-circulating strains (Gog and Grenfell 2002; Gomes et al. 2002; Gupta et al. 1996, 1998). Other studies have looked factors that can generate oscillations in strain incidence (Castillo-Chavez et al. 1989; Dawes and Gog 2002), or the frequency at which epidemics occur (Andreasen 2003).

Previous comparisons of strain models have generally focused on the dynamics of a small number of strains (Dawes and Gog 2002; Ferguson and Andreasen 2002; Ballesteros et al. 2009). However, one of the strengths of reduced frameworks is that they allow a much larger strain space to be explored. The price of this simplicity is usually information: it is often not possible to introduce a novel strain and use existing variables to calculate immunity against it (Table 1).

There are several biological questions which require effective tracking of population immunity. To examine whether a new strain can replace endemic strains, it is necessary to record the changing immune structure of the population in a tractable model. This can either be achieved by focusing on a small number of strains (Ballesteros et al. 2009), or by making simplifying assumptions about the cross-immunity function (Andreasen and Sasaki 2006; Boni et al. 2004).

History-based based models, which record the possible infection histories in a population, can also be compared with observed serological data to understand how individual- and population-level factors shape antibody responses over time (Kucharski and Gog 2012c). Using models that can calculate cross-immunity against unseen strains, it should also be possible to examine the introduction of novel strains similar to those that have previously circulated, as happened with the 2009 influenza pandemic (Miller et al. 2010; Xu et al. 2010).

## Incorporating pathogen evolution

### Stochastic emergence

New influenza strains emerge frequently through mutations with antigenic effects (Both et al. 1983). When modelling influenza evolution it is therefore necessary to consider the random nature of the mutation and emergence process, and integrate this with a model of the epidemic dynamics. In an individual-based model (Bedford et al. 2012; Ferguson et al. 2003; Tria et al. 2005), both processes can be modelled explicitly: within each infected individual, a strain may mutate with a certain probability, with the new infection either taking off or failing to emerge as a result of stochastic transmission in the population. However, emergence of new strains can also be included in the reduced frameworks described in the preceding sections.

As with choice of strain model, selecting an appropriate evolution framework depends on the biological dynamics of interest, and on the corresponding processes that are likely to be influenced by stochasticity. One study used a stochastic status-based model with evolution represented by an explicit genotype-to-phenotype map to investigate observed patterns of influenza diversity and strain replacement (Koelle et al. 2006). Alternatively, the evolutionary process can be implemented in a stochastic two-tiered model, with one tier representing population dynamics, and the other molecular evolution (Koelle et al. 2010). Such an approach makes it possible to model entire genetic sequences in a computationally viable way, and hence generate phylogenies that can be compared quantitatively with observed data.

If the information of interest is the speed of antigenic drift rather than the specific evolutionary trajectory of the virus, a simpler approach is to use a stochastic proxy for the evolution process, based on a probability distribution, along with a deterministic status-based (Koelle et al. 2009) or history-based (Minayev and Ferguson 2009) model for the epidemic. Such models assume mutation is stochastic, but do not include the possibility of extinction at start of an epidemic as the result of a stochastic transmission process. Branching processes can be used to approximate the stochastic transmission—or extinction—that can occur as a result of the small number of people initially infected with a new strain (Gog 2008; Kucharski and Gog 2012b). Such models assume that virus mutation is deterministic, but that transmission is initially stochastic when a new strain emerges.

### Separating epidemics from evolution

As there is evidence that temperate regions are annually ‘seeded’ with influenza after low levels of prevalence over the summer (Nelson et al. 2006), it is reasonable to consider the epidemic process during an influenza season separately from the evolutionary process between seasons.

Andreasen (2003) implemented annual epidemics as a discrete season-to-season map, which described the change in population immune structure as a result of antigenic evolution each summer following the winter outbreak. Cross-immunity in the model was assumed to reduce susceptibility, so if we assume births and deaths occur between seasons, the relevant part of the model given by Eqs. 15–16 each season is:

39 $$\begin{aligned} \frac{dS_{X}}{dt} = {}&\left\{ \! \begin{array}{l@{\quad }l} \tau (X {\setminus } i,i) \Lambda _i S_{X \setminus i} &{} \text {if } i \in X \\ - \tau (X,i) \Lambda _i S_{X} &{} \text {if } i \notin X \end{array} \right. \end{aligned}$$

40 $$\begin{aligned} \frac{d \Lambda _{i}}{dt} = {}&\beta \Lambda _i \sum _{i \notin Y}\tau (Y,i) S_Y - \gamma \Lambda _{i}. \end{aligned}$$

Next, define the expression

41 $$\begin{aligned} Q_i=\sum _{i \notin Y} \tau (Y,i) S_Y \end{aligned}$$

to be the ‘potential susceptibility’ to strain $i$. The model can be simplified by using a minimum function and assuming antigenic distance is always increasing. If $i$ is a new strain, with $i>j$ for each $j \in Y$, this means $\tau (Y,i) = \tau (\max Y,i)$. Let $\tilde{S}_j$ denote the set of individuals whose most recent infection was with strain $j$,

42 $$\begin{aligned} \tilde{S}_j=\sum _{\max Y=j} S_Y. \end{aligned}$$

Assuming that each epidemic starts and ends with a negligible level of infection ($0<I \ll 1$), we can find $p=Q_\infty /Q_0$, the ratio of final to initial potential susceptibility in season $i$, by solving $\log p + R_0 Q_0 (1-p)=0$, where $R_0=\beta /\gamma$. The value of $\tilde{S}_j$ at the end of the epidemic is

43 $$\begin{aligned} \tilde{S}_j(\infty )= {}&\left\{ \begin{array}{ll} (1-p) Q_0 &{}\quad \text {if } j =i \\ p^{\tau (j,i)} \tilde{S}_j(0) &{}\quad \text {if } j<i \end{array} \right. \end{aligned}$$

It is possible to perform a detailed bifurcation analysis in the case of two year immune recognition (Andreasen 2003), with immunity to a strain only conferring cross-immunity to strains that appear in the following two seasons, i.e. in the case

44 $$\begin{aligned} Q_i=\sum _{j=i-2}^i \tau (j,i) \tilde{S}_j. \end{aligned}$$

The season-to-season approach can also help simplify reduced transmission frameworks. In particular, by considering the age-structured multi-strain model in Eqs. 28–29 as a series of single epidemics, the system can be expressed using ODEs rather than PDEs (Kucharski and Gog 2012a). As the strains introduced each season are known, the sequence of possible infections that an individual could have seen is constrained by the order in which strains are introduced.

Further, under the single season approach evolution need not be independent of the epidemic process: Boni et al. (2004) explored a framework in which larger epidemics generated more antigenic drift, showing that a positive feedback can occur between the number of cases and number of new variants. Such a model can also be extended to examine the relationship between viral fitness and drift (Boni et al. 2006).

### Mutation-free approaches

It is also possible to model aspects of influenza dynamics without considering an explicit mutation process. Competition between strains resulting from cross-immunity has been seen to generate oscillations in disease incidence (Gupta et al. 1998; Lin et al. 1999) as well as sequential outbreaks of antigenically diverse pathogens (Recker et al. 2007). Further, the additional model tractability in the absence of a mutation process makes it possible to derive expressions for the conditions needed for invasion of new strains (Adams and Sasaki 2007, 2009) and transitions in epidemic dynamics (Blyuss and Gupta 2009; Blyuss 2012).

## Interface with data

### Infection and immunity

Tractable disease models have the advantage of being quick to simulate, which means they can be incorporated into inference frameworks. With the increasingly availability of detailed serological and social contact data (Conlan et al. 2010; Lessler et al. 2011; Mossong et al. 2008), multi-strain models are a promising tool with which to understand the processes behind infection and immunity for diseases such as influenza.

Exploring the age pattern of immunity to seasonal influenza with such models, it has been shown (Kucharski and Gog 2012c) that observed data are best explained with a model that uses physical contacts and incorporates the phenomenon of ‘original antigenic sin’, whereby the first infection of a lifetime inhibits subsequent acquisition of immunity (Francis 1960). To model original antigenic sin, it is necessary to know the order of the strains a host’s infection history; in particular, the antigenic properties of the first infection of a lifetime. Such information is available for a single season model, as the framework is designed so that one specific strain is introduced each year (Andreasen 2003). However, it would less straightforward to keep track of strain order if multiple strains were co-circulating.

Recent empirical studies have suggested a pattern of ‘antigenic seniority’ for influenza, with antibody titres higher to ‘senior’ strains seen earlier in life (Lessler et al. 2012; Miller et al. 2013). It has also been noted that elderly individuals have fewer naive B cells (Weinberger et al. 2008). There may well be a unifying mechanism behind these disparate observations; identifying it would greatly improve our understanding of how populations build immunity to diseases like influenza over the course of a lifetime.

The interaction of different strains through cross-immunity can also be examined using disease case data. Inference methods based on Sequential Monte Carlo algorithms have recently been developed for two-strain models (Shrestha et al. 2011); the natural next step would be to scale up this approach to explore a larger number of strains.

### Other pathogens

Many multi-strain modelling studies have focused on influenza (Andreasen et al. 1997; Gog and Grenfell 2002; Ferguson et al. 2003), *Plasmodium falciparum* (Gupta and Day 1994; Gupta et al. 1994) or *Neisseria meningitidis* (Gupta et al. 1996; Buckee et al. 2008), but high-dimensional frameworks are likely to be relevant to other pathogens as well. Phylogenetic analysis has shown that dengue viruses (DENV) can be subdivided into four distinct groups (Grenfell et al. 2004), known as serotypes. When primary infection with one DENV serotype is followed by a secondary infection with a different DENV serotype, it can result in severe disease. It has been suggested this is caused by ‘antibody-dependent enhancement’, with prior immunity promoting rather than suppressing replication of the second virus (Dejnirattisai et al. 2010).

Models with up to four strains have previously been used to examine the effects of antibody-dependent enhancement on epidemic patterns (Nagao and Koelle 2008; Recker et al. 2009; Wikramaratna et al. 2010) and serotype diversity (Ferguson et al. 1999; Kawaguchi et al. 2003; Cummings et al. 2005). However, DENV shows evidence of genetic variation within serotype, and prior infection with different variants does not always result in the same response to a subsequent infection (Watts et al. 1999). The severity of disease is therefore likely determined by both genetic variation and serotype-specific immunity (Ohainle et al. 2011). High-dimensional strain models have been used to address a number of questions about influenza; interfaced with appropriate data, they would also be a natural tool with which to explore DENV.

### Evolutionary dynamics

Multi-strain models can also be a useful tool for exploring disease ‘phylodynamics’: the interaction between pathogen evolution and population-level transmission (Grenfell et al. 2004). It can be challenging to perform statistical inference with such frameworks, however, as model complexity often means it is not possible to derive a likelihood function that incorporates both genetic and population-level data.

One solution is to focus on simple transmission models. For example, statistical inference for seasonally-forced SIR models can be performed using disease case data and sequence data (Rasmussen et al. 2011). Using techniques such as particle Markov chain Monte Carlo (Andrieu et al. 2010), such frameworks can be used to estimate key epidemiological parameters. Alternatively, approximate Bayesian computation (ABC) can be used to compare multi-strain models with case reports and sequence data when the likelihood function is intractable (Ratmann et al. 2012).

**Fig. 4:**
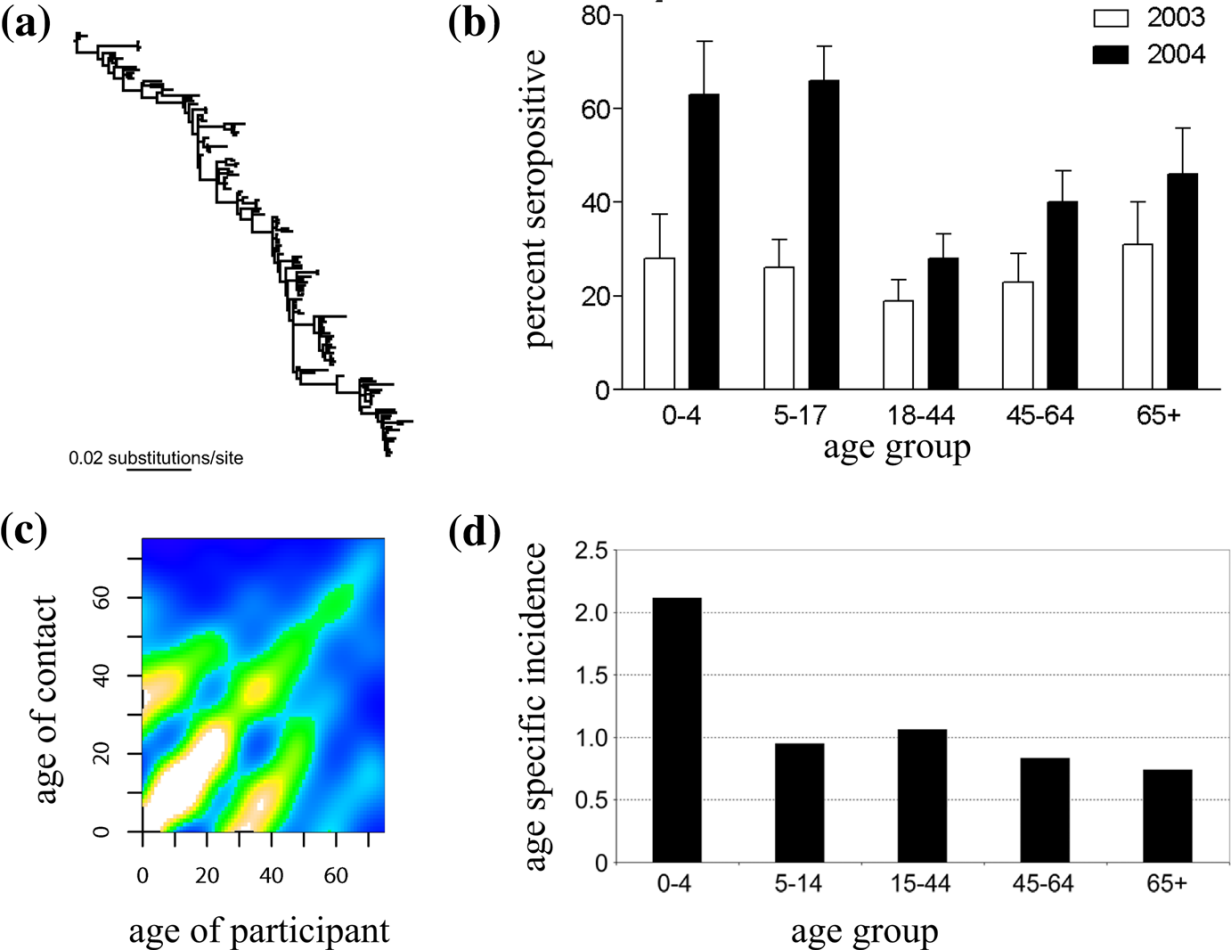
Potential sources of data. **a** Phylogenetic tree for influenza subtype H3N2 [adapted from Holmes and Grenfell (2009)]; **b** percent of sampled individuals in Britain with immunity to 2003 H3N2 strain in 2003 and 2004 [adapted from Johnson et al. (2009)]; **c** results of contact survey in Great Britain [adapted from Mossong et al. (2008)], with lighter colours representing a larger number of reported contacts between those age groups; **d** age specific incidence of ILI, as factor of all-age incidence, for 2003/4 influenza season in Britain [adapted from Johnson et al. (2009)]

Such studies have focused on genealogies and disease incidence, without incorporating data on population structure or serological data in their inference frameworks. However, it has been observed that strain diversity can increase when a contact network (Buckee et al. 2004) or community structure (Buckee et al. 2007) is introduced to a multi-strain model. Further, there is not a clearly defined relationship between antigenic and genetic variation for pathogens such as influenza (Smith et al. 2004). As such, reported social contacts, serological surveys, disease incidence and virus isolate sequences (Fig. 4) may all need to be considered in the analysis of antigenically variable pathogens.

Combining such data in a modelling framework presents a number of theoretical challenges. First, to translate model variables into quantities that can be measured empirically, multi-strain models need to be combined with an observation process. For example, given the increasingly availability of serological data, combining a statistical model of antibody titres with a transmission model would make it possible to investigate population level dynamics using data on individual-level titres. Second, we need to examine the factors that influence evolution at different temporal and spatial scales. In particular, models could be used to explore how selection pressure acts on a virus both in terms of bottlenecks during transmission between hosts and the background of prior population immunity. Models of multi-strain pathogens could also be used to understand how within-host immune dynamics influence the acquisition of immunity over a lifetime, and hence the evolutionary trajectory of a disease. By developing such models in a way that makes them easily compatible with data, there is potential to substantially improve our knowledge of how population structure, evolution and immunity contribute to observed disease dynamics.
